# Simulated microgravity modulates the mesenchymal stromal cell response to inflammatory stimulation

**DOI:** 10.1038/s41598-019-45741-8

**Published:** 2019-06-26

**Authors:** Andrey Ratushnyy, Danila Yakubets, Elena Andreeva, Ludmila Buravkova

**Affiliations:** 0000 0004 0390 4822 grid.418847.6 Lab. of Cell Physiology, Institute of Biomedical Problems of Russia Academy of Sciences, Moscow, 123007 Russia

**Keywords:** Mechanisms of disease, Immunochemistry

## Abstract

The duration and distance of manned space flights emphasizes the importance of advanced elucidation of space flight factors and their effects on human beings. The exposure to inflammatory mediators under microgravity may contribute to the activity of different cells, perivascular stromal cells (MSCs) in particular. Inflammatory activation is now considered as a principal cue of MSC engagement in reparative remodeling. In the present paper, the effect of simulated microgravity (sµg) on TNFα-mediated priming of adipose tissue-derived MSC (ASCs) was examined. Sµg *per se* did not induce inflammatory-related changes, such as elevation of ICAM-1 and HLA-ABC expression, soluble mediator production, or shifting of the transcription profile in ASCs. Moreover, the attenuated ASC response to TNFα priming under sµg was manifested in decreased production of TNFα-dependent pleiotropic cytokines (IL-8 and MCP-1), matrix remodeling proteases, and downregulation of some genes encoding growth factors and cytokines. Time-dependent analysis detected the first signs of priming attenuation after 48 hours of 3D-clinorotation. A reduced response of MSCs to priming under sµg can be a negative factor in terms of MSC involvement in tissue remodeling processes.

## Introduction

Space flight factors, such as microgravity, space radiation, etc., pose serious risks to the health of astronauts who spend increasing lengths of time on board manned space vehicles. The results of space flight and gorund-based  experiments suggest that microgravity causes evident negative changes in the cardiovascular system^1–3^ and bone tissue^4–7^. Adaptation of the vasculature to various extreme factors is determined primarily by the vessel wall state, i.e., endothelial cells (ECs), and the surrounding perivascular cells that are now commonly considered to be multipotent mesenchymal stromal cells (MSCs)^8^. The vascular wall cells are known to be mechanosensitive. They react to physical factor changes (including gravity). This reaction can result in the development of cardiovascular dysfunction.

Space experiments and ground-based studies have demonstrated that ECs are very sensitive to modulation of a gravitational stimulus, demonstrating the signs of endothelial dysfunction^9–11^. Opportunities of studying the EC monolayer behavior under real microgravity are extremely limited; therefore, the data obtained in ground-based experiments are one of the most important sources of knowledge on the mechanisms of EC adaptation to microgravity. To simulate the effects of microgravity on adherent cells, 2D- and 3D-clinorotation (random positioning machine (RPM)) are considered the most appropriate^12,13^. These models have shown that ECs demonstrate both early and delayed responses^9–11,14,15^. Exposure to adverse microenvironmental factors (such as inflammatory mediators) under microgravity may contribute to deterioration of the vascular wall condition. In an inflammatory response, leukocytes are known to transmigrate through the endothelial lining from the bloodstream to the adjacent tissues^16^. According to the findings of Griffoni *et al*.^17^ and our laboratory^18^, microgravity *per se* is not an inflammatory stimulus for ECs. Under inflammatory activation, sµg could supposed to enhance the increase in leukocyte adhesion and transmigration, thereby modulating EC dysfunction. In fact, we have recently demonstrated that sµg potentiates the effect of EC activation by inflammatory mediators but does not affect the expression of adhesive cascade molecules on the ECs^18^.

The effect of microgravity on cells of the stromal lineage, MSCs in particular, is currently being considered primarily due to their potential for differentiation, production of soluble mediators, and involvement in bone tissue remodeling^19,20^. A series of studies on the effects of sµg on human bone marrow stromal lineage cells of different commitment have been performed. The revealed structural and molecular alterations confirmed the existence of gravity-dependent intracellular mechanisms that cause both early and late stromal progenitor cell responses to sµg. These findings have expanded the current views on the mechanisms of adult progenitor cell susceptibility to changes in the gravitational environment, at least *in vitro*, and on the role of the actin cytoskeleton as a gravity-sensitive structure. The susceptibility/resistance of MSC to inflammatory stimuli under sµg is poorly studied. At the same time, there is a significant set of data indicating the important role of inflammatory priming in MSC functional activity^21^. This effect has been studied in the most detail regarding induction of the immunosuppressive potential of MSCs^22^. Meanwhile, the alteration of proliferation, differentiation, hematopoiesis-supportive, and paracrine activities of MSCs may occur in response to the factors released by activated immune cells.

In this study, we investigated how ASCs respond to TNFα inflammatory activation under sµg.

## Results

### ASC viability and growth under different experimental conditions

Neither priming nor sµg had a significant impact on ASC viability and growth. The proportion of living cells (PI-) in the AnnV-PI test did not differ between the experimental groups and was more than 95%. With an initial seeding density of 3,000 cells/cm^2^, the cell monolayer densities at the end of the experiment were similar, averaging 13,800 ± 3,100 cells/cm^2^ in the control (no exposure), 13,440 ± 5,000 under sµg, 15,300 ± 5,500 with TNFα, and 15,700 ± 5,900 under sµg with TNFα activation (data are presented as M ± SD). Thus, the changes in the levels of paracrine mediators in the conditioned medium shown below were not due to intergroup differences in cell growth or cell death.

### Effect of TNFα on ASC functions

**Figure 1:**
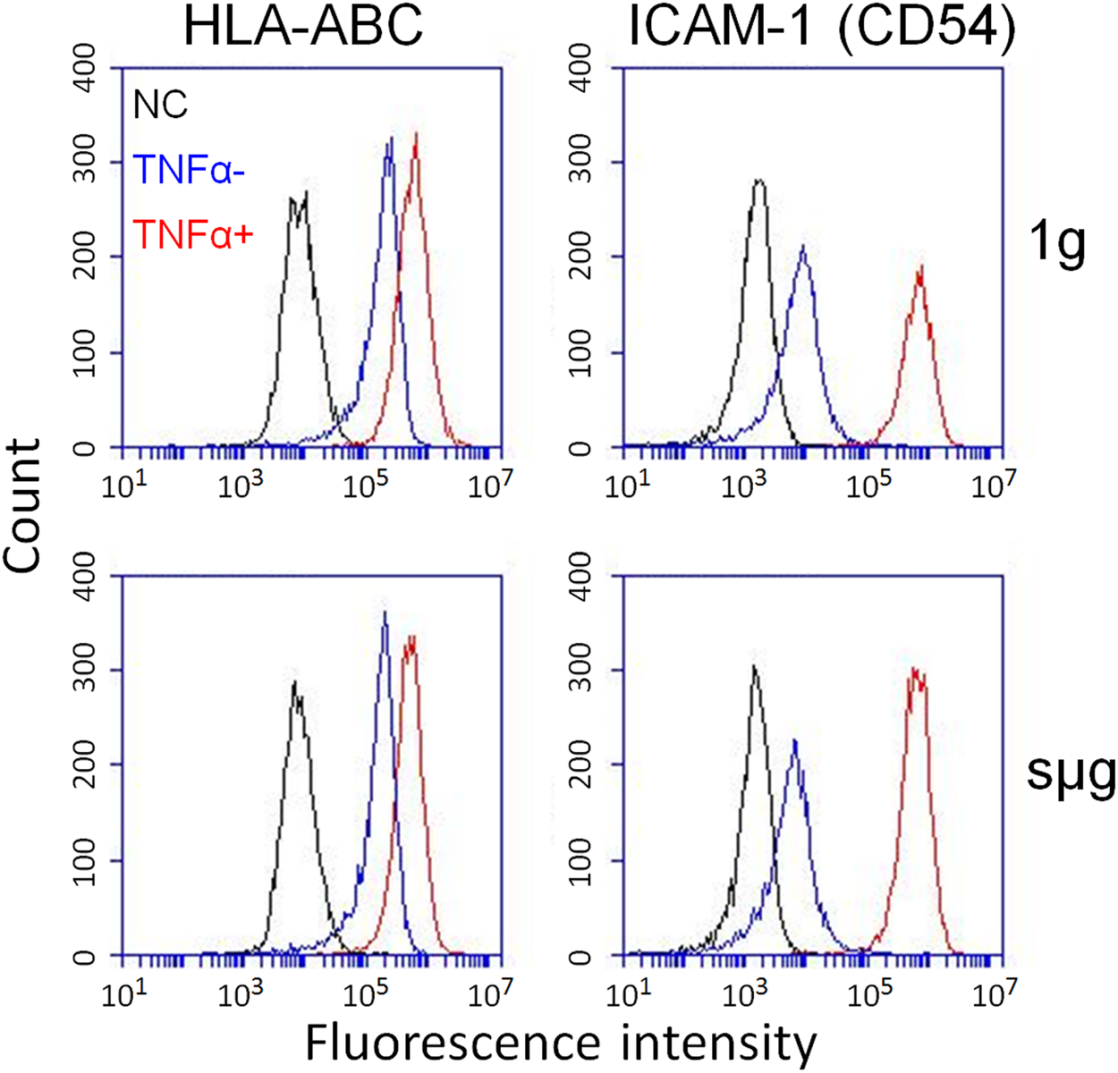
The effect of TNFα-mediated priming and sµg on the expression of inflammatory markers on ASCs. Representative flow cytometry histograms. ASCs were stained with antigen-specific fluorescent antibody against ICAM-1 and HLA-ABC. NC – negative control. ASCs were stained with matched fluorescent nonimmune antibody. TNFα+/TNFα− -ASCs were cultured with/without TNFα. sµg/1 g - simulated microgravity/control.

**Figure 2:**
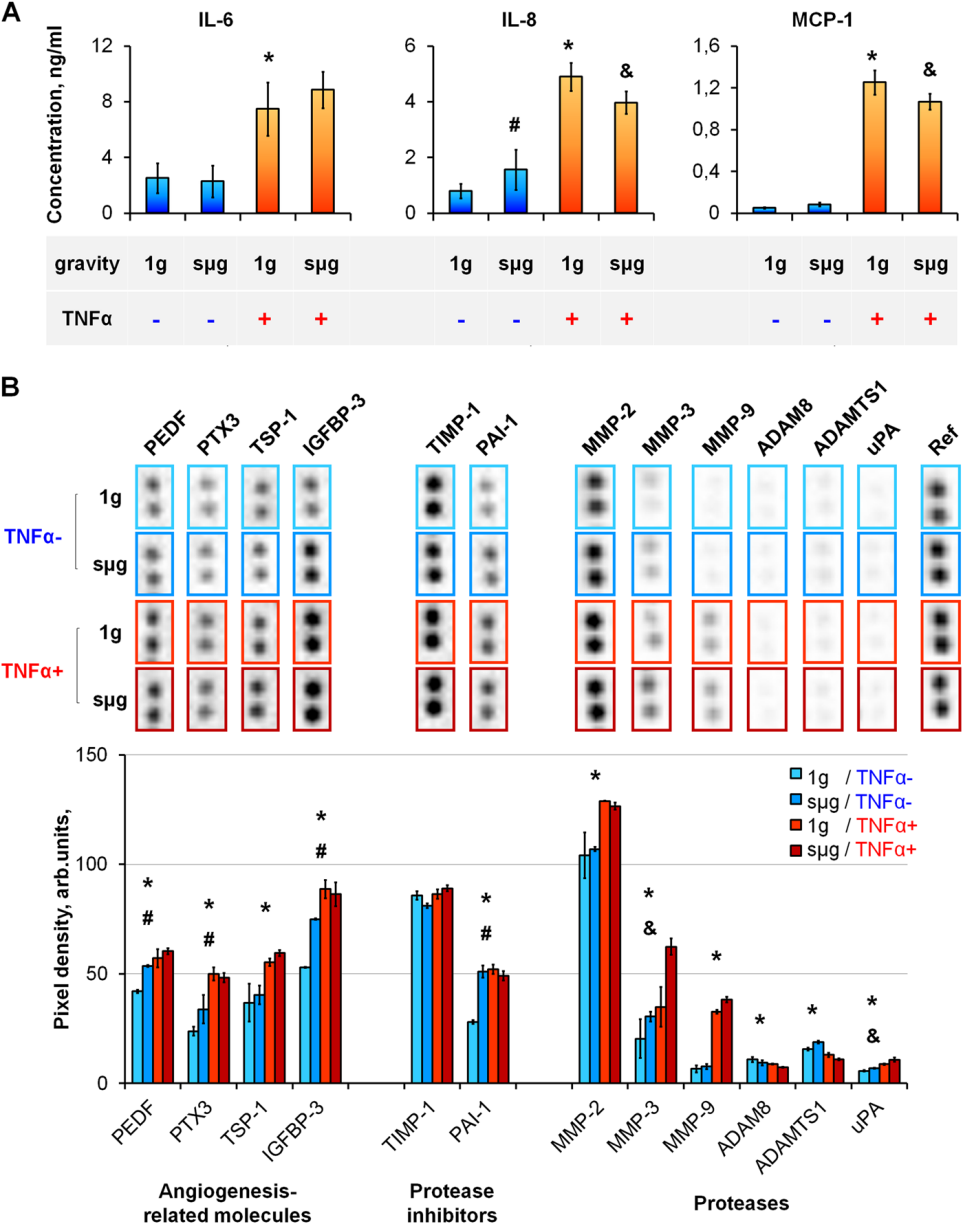
The effect of TNFα-mediated priming and sµg on paracrine activity of ASCs. (**A**) Production of pleiotropic inflammatory cytokines. (**B**) Proteases and their inhibitors associated with inflammatory activation. Representative dot-blot and average level in conditioned medium. The data are expressed as mean ± standard deviation (n ≥ 3); ^*,**#**,&^p < 0.05. *1 g/TNFα+ vs 1 g/TNFα−; ^**#**^sµg/TNFα− vs 1 g/TNFα−; ^&^sµg/TNFα+ vs 1 g/TNFα+.

Single inflammatory mediators (TNFα, IFNγ, IL1β) as well as the mediator cocktail produced by activated immune cells, are known to provoke a change in the expression profiles of MSC surface antigens, the inducible ICAM-1 molecule (CD54), and the main histocompatibility complex class I HLA-ABC molecules in particular^23,24^. In this study, ASCs were exposed to TNFα for 96 hours. Flow cytometry detected 100- and 3-fold increases in the mean fluorescence intensity (MFI) of ICAM-1 and HLA-ABC, respectively, which confirmed priming (Fig. 1). TNFα exposure stimulated the production of the main pro-inflammatory cytokines. It was detected 3-fold increase of IL-6, 6-fold increase of IL-8, and 24-fold increase of MCP-1 in ASC conditioned medium (Fig. 2A).

Using dot-blot analysis, the enzymes associated with inflammatory activation and their inhibitors were determined in the conditioned medium (Supplementary Fig. S1). After a 96-hour exposure, the levels of metalloproteases increased significantly (ММР-2, ММР-3, ММР-9), but the level of metalloprotease inhibitor TIMP-1 remained unchanged. At the same time, the levels of disintegrin and metalloprotease family with thrombospondin motif enzymes (ADAM8, ADAMTS1) decreased. The levels of urokinase (uPA) and its inhibitor (plasminogen activator inhibitor-1, PAI-1) increased significantly. These findings make it possible to conclude that inflammatory activation provoked a significant alteration in the profiles of the main enzymes involved in extracellular matrix remodeling (Fig. 2A,B).

TNFα priming has been previously shown to increase MSC production of growth factors, including angiogenic ones, and other biologically active molecules, as well as transcription of their encoding genes^25^.

**Figure 3:**
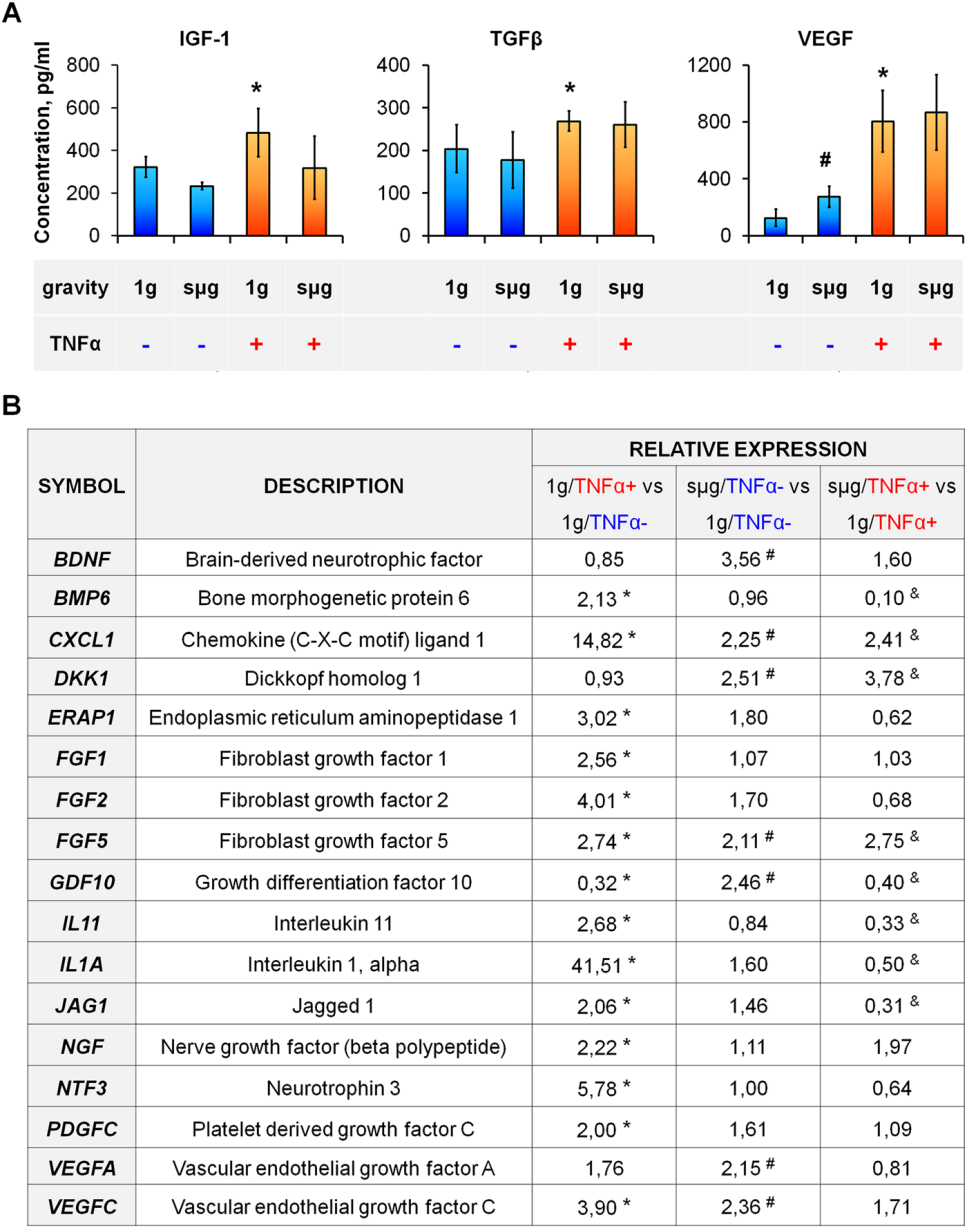
The effect of TNFα-mediated priming and sµg on ASC growth factors. (**A**) Growth factor production. The data are shown as mean ± standard deviation (n ≥ 3). (**B**) Differential gene expression. The data are shown as mean of relative expression (n ≥ 3). ^*,**#**,&^p < 0.05. ^*^1 g/TNFα+ vs 1 g/TNFα−, ^#^sµg/TNFα− vs 1 g/TNFα−, ^&^sµg/TNFα+ vs 1 g/TNFα+.

After TNFα exposure, we have demonstrated  a significant elevation of some ASC growth factors: pigment epithelium-derived factor (PEDF) (Fig. 2B), insulin-like growth factor-1 (IGF-1), transforming growth factor β (TGF-β) (Fig. 3A). In addition, increased levels of biologically active molecules that regulate angiogenesis were noted: TNF-inducible pentraxin-related protein 3 (PTX-3), thrombospondin-1 (TSP-1), and IGF-binding protein-3 (IGFBP-3) (Fig. 2B) as well as the main angiogenic mediator VEGF (Fig. 3A).

Transcriptome profiling of ASCs after TNFα exposure demonstrated significant upregulation of genes encoding growth factors and related molecules: *FGF1*, *FGF2*, *FGF5*, *NGF*, *NTF3*, *PDGFC*, and *VEGFC* (Fig. 3B). In addition, cytokine *IL1A*, *IL11*, and *CXCL1* and endoplasmic reticulum aminopeptidase *ERAP1* genes were upregulated. The only downregulated gene was *GDF10* belonging to the TGF-β superfamily that is closely related to bone morphogenetic protein-3 (BMP-3). Such changes in the levels of gene expression confirmed the effect of inflammatory activation of ASCs (Fig. 3B, column 1 g/TNFα+ vs 1 g/TNFα−).

### Effect of sµg on ASC functions

To assess the effects of sµg on the ASC response to an inflammatory stimulus, it was necessary to understand whether the sµg *per se* influenced the parameters of inflammatory activation of ASCs described above.

According to flow cytometry, RPM exposure had no effect on the expression of ICAM-1 and HLA-ABC molecules (Fig. 1). Analysis of soluble mediators in the conditioned medium found a slight but significant increase in the production of IL-8 (Fig. 2A) and VEGF (Fig. 3A); the levels of proteases remained almost unchanged.

Seven genes were upregulated in ASCs after RPM exposure. It was two times less in comparison with TNFα priming (Fig. 3B). Only four genes were the same in both groups: *CXCL1*, *FGF5*, *VEGFC*, and *GDF10*. Transcription of *CXCL1*, *FGF5*, and *VEGFC* was elevated under sµg, though to a lesser extent. *GDF10* was also upregulated, in contrast to its downregulation in primed ASCs. In sµg-ASCs, *BDNF* and *VEGFA* genes were overexpressed in contrast to TNFα-primed ASCs. Thus, sµg had almost no effect on the parameters altered in ASCs with TNFα priming (Fig. 3B, column sµg/TNFα− vs 1 g/TNFα−).

### Pro-inflammatory activation of ASCs under simulated microgravity

Exposure under sµg had no effect on the TNFα-induced elevation of ICAM-1 and HLA-ABC expression (Fig. 1). The viability and growth efficiency of ASCs also did not differ versus other experimental groups.

ASCs primed under sµg produced significantly less of the major pleiotropic cytokines IL-8 and MCP-1 (Fig. 2A). Comparison of the transcriptional activity of ASCs primed in the static control and under sµg demonstrated downregulation of several genes under sµg: *BMP6*, *GDF10*, *IL11*, *IL1A*, and *JAG1* (Fig. 3B, column sµg/TNFα+ vs 1 g/TNFα+). Thus, the ASC response to an inflammatory stimulus was attenuated under sµg.

### Time-dependent changes in ASC mediator production under TNFα and sµg exposure

In the previous section, a decreased efficiency of ASC priming under sµg compared to static control priming after 96 hours of exposure was demonstrated. These changes were manifested primarily in a decreased production of paracrine mediators. To determine how rapidly the effect of inflammatory priming attenuation appeared, cytokine levels and the expression of the corresponding genes were investigated at earlier time points (6, 24 and 48 hours).

**Figure 4:**
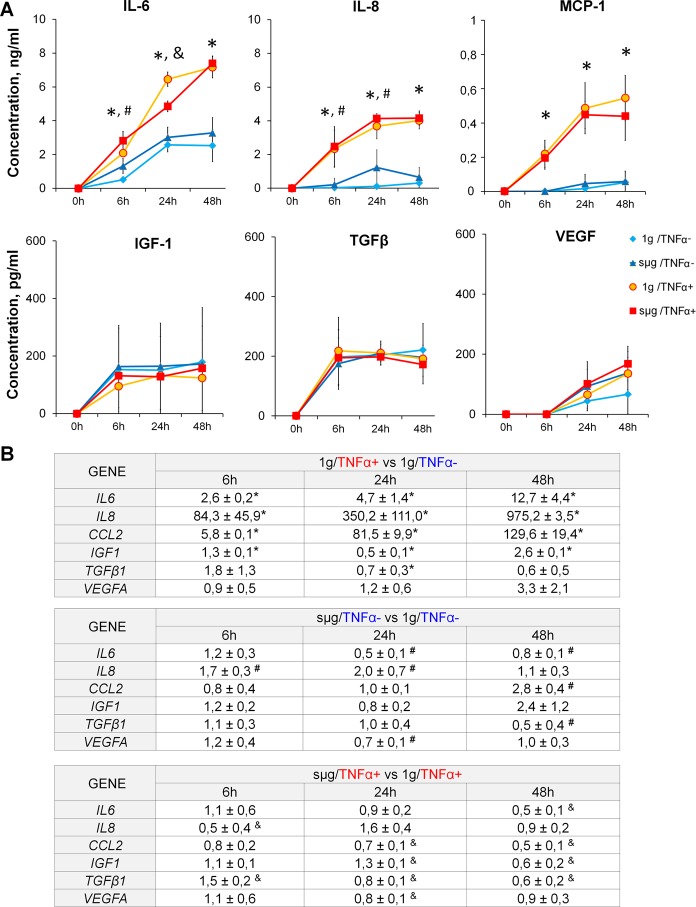
Time-dependent analysis of interleukins and growth factors secretion and differential gene expression in ASCs after TNFα-mediated priming and sµg exposure. (**A**) Soluble mediator production. The data are shown as mean ± standard deviation (n ≥ 4). (**B**) Differential gene expression. The data are shown as mean of relative expression ± standard deviation (n ≥ 4). ^*,#,&^p < 0.05. ^*^1 g/TNFα+ vs 1 g/TNFα−, ^**#**^sµg/TNFα− vs 1 g/TNFα−, ^&^sµg/TNFα+ vs 1 g/TNFα+.

The pleiotropic cytokines IL-6, IL-8, and MCP-1 increased in all experimental groups, reaching a plateau by 24 hours, on average. During TNFα stimulation, the levels of these cytokines were significantly higher. After 24 hours, cytokine production remained almost unchanged (Fig. 4A). At the same time, the dynamics of gene expression differed between the compared groups. Under TNFα priming, *IL8*, *CCL2*, and *IL6* increased linearly over 6 to 48 hours (р < 0.05). After RPM exposure, significant upregulation was detected only for *IL8* after 24 hours and for *CCL2* after 48 hours. No changes in the activity of cytokine genes were found in ASCs primed under sµg, except of a downregulation of *CCL2* after 48 hours (p < 0.05) (Fig. 4B).

Regardless of the experimental conditions, the levels of IGF-1 and TGF-β1 in the conditioned medium reached a plateau after as early as 6 hours of culture. VEGF levels increased linearly up to 48 hours. Significant changes in the transcription of the corresponding genes were noted after 48 hours only (Fig. 4A). Under sµg, *IGF1* was upregulated, while *TGFB1* was downregulated. With priming, only *VEGFА* expression was increased (Fig. 4B). Compared to ASC priming under static conditions, these effects were canceled under sµg. Thus, at the early stages of exposure, sµg has almost no effect on the production of TNF-induced inflammatory cytokines and the growth factors examined, as well as on the transcription of the corresponding genes.

## Discussion

Dysregulation of various cell system functions is one of the main negative effects caused by space flight factors. These changes may occur due to alterations in the paracrine profile, particularly those associated with increased plasma levels of regulatory cytokines in astronauts. Thus, elevations in TNFα, IL-8, IL-1ra, thrombopoietin (Tpo), VEGF, MCP-1, chemokine ligand 4/macrophage inhibitory protein 1b (CCL4), and C-X-C motif chemokine 5/epithelial neutrophil-activating protein 78 (CXCL5) were associated with spaceflight^26^. Increased inflammatory cytokines can significantly affect the functional activity of stromal progenitors, MSCs in particular.

Inflammatory activation (priming) of MSCs is one of the most important mechanisms by which the functional activity of these cells is involved in reparative tissue remodeling^21^. In the presence of both inflammatory cytokines and activated immune cells, MSCs start the synthesis of immunosuppressive mediators that are not produced in the steady state (inducible cyclooxygenase-2, COX-2) and upregulate constitutive anti-inflammatory metabolites (IDO, a key T-cell-proliferation-suppressing enzyme)^27,28^. In addition, priming affects the MSC functions not associated with immunosuppression. To date, MSCs are known to retain their characteristic mesenchymal phenotype (SSEA4, CD73, CD90, CD105, CD29, CD44) and the capacity for mesenchymal multilineage differentiation upon exposure to inflammatory cytokines^23,29^. The proliferative activity and cytokine profile of MSCs, as well as their ability to migrate, vary depending on the cytokine used^23,30^. Recently, it has been shown in our laboratory that the cocktail of inflammatory mediators produced by mitogen-stimulated immune cells causes a significant upregulation of ASC genes involved in inflammatory activation, immunosuppression, proliferation, regulation of cytokine production, extracellular matrix remodeling, etc. As well, increased mitochondrial transmembrane potential, decreased endoplasmic reticulum volume, and an increased level of ICAM-1 expression were observed^24^. Direct contact of MSCs with activated immune cells potentiated the effect of the inflammatory cocktail, causing a decrease in the proliferative and migratory activities of MSCs, as well as a reduction of the differentiation potential^31^.

As for the effect of sµg on MSCs, RPM exposure has been shown to result in significant changes in the bone marrow MSC cytoskeleton, decelerated proliferation, reduced osteogenic commitment, and altered transcriptional activity of a number of genes responsible for various biological processes^32,33^. In experiments with adipose tissue perivascular MSCs (ASCs), we demonstrated decreased expression of a number of adhesion-associated proteins and altered transcriptional activity of genes encoding adhesion proteins and matrix^34^. A modified secretory profile and the resulting increase in the angiogenic potential of ASCs have been shown^35^.

In the present study, we demonstrated a reduced ASC response to an inflammatory stimulus under sµg, which was manifested in the decreased production of TNFα-dependent IL-8 and MCP-1 pleiotropic cytokines, matrix remodeling proteases, and downregulation of genes encoding growth factors and cytokines. According to time-dependent dynamics analysis, the first signs of attenuation of priming efficacy occurred after 48 hours of RPM exposure. At 96 hours, the changes in most of the parameters analyzed reached a plateau.

Unfortunately, no published data regarding MSC priming under sµg are available; therefore, to understand the features of the cell response to an inflammatory stimulus under deprivation of gravity, one should refer to the findings obtained with other cell types.

In experiments using human endothelial cells, we have demonstrated that the combined effects of pro-inflammatory activation and microgravity cause more significant damage, resulting in the destruction of stress fibers, microfilament depolymerization, and potentiation of TNFα-induced upregulation of adhesion molecules and adhesive contact genes (*ICAM1*, *VCAM1*, *CDH5*, *IL8*), and elevation of IL-8 production^18^. To elucidate the probable mechanisms of combined effect of inflammatory activation and sµg it will be useful to consider the data of recent studies^36,37^. It is known that bFGF is elevated under inflammatory TNFα exposure. It has been shown that bFGF secretion was attenuated, while the exogenous bFGF was less effective in stimulation of accumulation of soluble TNFRSF5, TNFSF5, intercellular adhesion molecule-1, TNF- receptor 2 in EC supernatants under sµg^36^. According to Ulbrich *et al*. (2008), exogenous bFGF provoked elevation of fibronectin, Flk-1 and Flt-1 proteins, and reduction of IL-6 and IL-8 in sµg-exposed ECs compared with vehicle treated sµg cells. Also, bFGF at sµg contributed to protect endothelial cells from apoptosis^37^. Thus, the combined effects of inflammatory stimulus and gravity deprivation may be realized through the growth factor-regulated signaling cascades.

The results of LPS activation of mouse macrophages under sµg manifested in decreased TNFα production may be an example of the negative microgravity effect on the inflammatory response. Analysis of molecular mechanisms demonstrated no changes in NF-κB pathway activity but indicated significant upregulation of heat shock factor-1 (HSF-1), the known TNFα promoter repressor^19^.

Analysis of the effects of IL-6 superfamily belonging oncostatin M (OSM), on MC3T3-E1 osteoblasts in the Rotary Cell Culture System (RCCS) revealed that OSM and the RCCS independently and synergistically induced IL-6 production. In 12 hours, IL-6 production increased 7-fold after OSM exposure, 2-fold after RCCS exposure, and 350-fold after a combined exposure. At the same time, independent and multidirectional regulation of functional osteoblast activity was observed with inflammatory OSM and RCCS microgravity exposure. OSM supplementation upregulated collagen α1(I) and osteocalcin genes but had no significant effect on sclerostin transcription. RCCS downregulated collagen α1(I) and osteocalcin while upregulating sclerostin^38^.

Thus, depending on the particular inflammatory inducer, microgravity model, and cell type, the results of inflammatory priming can vary significantly. Why is this important for perivascular MSCs? The levels of the main inflammatory mediators, TNFα and IFNγ, are known to determine MSC phenotype polarization^39^. At a high concentration, a shift occurs to enhance anti-inflammatory, including immunosuppressive, activity (MSC2). In contrast, with a low level of inflammatory mediators, MSCs acquire an inflammatory phenotype (MSC1) and, as a result, trigger the activation of immune cells. The increased levels of TNFα and IL-8 were observed in spaceflight recently^26^, supposing that anti-inflammatory activation of MSCs could be occurred in a proper fashion. Meanwhile, a reduced response of MSCs to priming under sµg can be a negative factor in terms of MSC involvement in tissue remodeling processes. These data can help in the analysis of the peculiarities of inflammatory reactions in human beings under microgravity and stimulate the development of new approaches to the treatment of pathological conditions in long-term space flights.

## Methods

All methods were performed in accordance with the relevant guidelines and regulations of this journal.

### Isolation and culture of adipose-tissue‐derived mesenchymal stromal cells

Adipose tissue samples were obtained under the Scientific Agreement from Soyuz multidisciplinary clinic (Moscow, Russia) after elective liposuction procedures performed with local anesthesia from healthy patients with written informed consent. Adipose tissue was processed using the guidelines specifically approved by the Biomedicine Ethics Committee of the Institute of Biomedical Problems, Russian Academy of Sciences (Physiology Section of the Russian Bioethics Committee, Russian Federation National Commission for UNESCO, Permit #314/МCK/09/03/13). Adipose stromal cells (ASCs) were isolated using a standard method described by Zuk *et al*.^40^ as modified by Buravkova *et al*.^41^. The cells were expanded in α‐MEM (Gibco, Life Technologies, USA) with 50 U/ml penicillin‐streptomycin (PanEco, Russia), and 10% fetal bovine serum (FBS) (HyClone, USA) at standard conditions (5% CO_2_, 37 °C).

The isolated cells were stained with antibody against stromal markers CD90, CD73, and CD105 and were analyzed using an Accuri C6 flow cytometer (BD Biosciences, USA). To induce osteogenic differentiation, complete α-MEM was supplemented with 10^−8^ М dexamethasone, 10 mМ glycerol-2-phosphate, and 0.2 mМ L-ascorbic acid 2-phosphate (Sigma, USA). Osteogenic differentiation was confirmed with Alizarin red staining of the mineralized matrix components (Millipore, USA). To induce adipogenic differentiation, the medium was supplemented with 0.5 mМ isobutyl methylxanthine, 1 μM dexamethasone, 10 μg/ml insulin, and 200 μM indomethacin (Sigma, USA). Adipogenic differentiation was assessed by the evaluation of cytoplasmic Oil-Red-O-stained lipid droplets (Millipore, USA).

ASCs were complied with the minimal set of criteria of a joint IFATS and ISCT statement^42^. Briefly, ASCs were adhered fast and displayed a fibroblast-like morphology. More than 95% of ASCs were positively stained with antibody against stromal markers CD90, CD73, and CD105, as demonstrated by flow cytometry. ASCs underwent osteo- and adipogenic differentiation in the presence of the appropriate stimuli in the medium.

### Simulated microgravity and TNFα priming

A desktop random positioning machine (RPM) (Dutch Space, Leiden, the Netherlands) was used to simulate the effects of microgravity. The speed (53–65 deg/s) and direction of the device rotation were randomized by dedicated control software at the computer user interface. The maximum distance between the cell monolayer and the center of rotation was 7.5 cm. The gravity value averaged 10^−2^ g^12^.

The cells were plated in a culture flask (surface area: 25 cm^2^, volume: 50 ml, Cellstar, Greiner Bio-One, Germany) at a density of 3,000 cells/cm^2^ 4 days before the start of the experiment. After ASCs reached 80–90% confluence, culture flasks were completely filled with medium without air bubbles to prevent sloshing of the medium and shear stress. The RPM with flasks fixed on the desktop was placed in a thermostat under standard temperature conditions at 37 °C. Samples were exposed on the RPM for 6, 24, 48, and 96 hours. Another group of flasks was placed in a routine CO_2_ incubator (37 °C), to serve as a control. The cells in half of the flasks in both groups were activated with TNFα (2 ng/ml). Inflammatory activation was confirmed by surface expression of ICAM-1 (CD54) and HLA-ABC in ASCs. Cells were stained with antibodies against ICAM-1 (CD54) and HLA-ABC and analyzed by flow cytometry (Accuri C6, BD Biosciences, USA). After exposure, conditioned medium from all samples was collected for further analysis, the cells were washed with PBS, and total RNA was extracted for analysis of gene expression by qPCR.

### ASC viability and growth

To study ASC viability, cells were stained with annexin and propidium iodide. ASCs were trypsinized, and the suspension was stained with an Annexin V–FITC kit (Immunotech, France) according to the manufacturer’s instructions. Cells were analyzed using an Accuri C6 flow cytometer.

Cell density was evaluated using the following equation: N_96_/SA, where N_96_ was the number of cells at the end of the experiment (at 96 h of exposure), and SA was the flask surface area (25 cm^2^). The cell numbers were determined using a hemocytometer.

### Analysis of proteins secreted by ASCs

To characterize the paracrine activity of ASCs, conditioned medium was collected, centrifuged at 2,500 *g* to remove cell debris, and stored at –70 °C until the measurements were taken. To detect secreted proteins, conditioned medium was analyzed using the Proteome Profiler Human Angiogenesis Array Kit (R&D, USA) and Proteome Profiler Human Protease Array Kit (R&D, USA), according to the manufacturer’s instructions. The data were analyzed using Image Lab™ Software Version 5.0 (Bio-Rad, USA). VEGF-α, TGF-β, IL-6, IL-8, MCP-1, and IGF-1 concentrations in ASC conditioned medium were evaluated using the Human VEGF ELISA Set (Peprotech, USA), Human TGF-β1 DuoSet ELISA (R&D, USA), Human IL-6 ELISA Set (BD, USA), Human IL-8 ELISA Set (BD, USA), Human CCL2/MCP-1 DuoSet (R&D, USA), and Human IGF-1 DuoSet (R&D, USA), according to the manufacturer’s instructions.

### Quantitative PCR analysis

To evaluate gene expression, total RNA was extracted with QIAzol Reagent (Qiagen, USA) and purified by the phenol/chloroform technique. The quality and concentration of RNA samples were estimated by using a Nanodrop ND-2000c (Thermo Scientific, USA). Reverse transcription was performed using the QuantiTect Reverse Transcription Kit (Qiagen, USA) according to the manufacturer’s protocol. Expression of 84 growth factor genes was analyzed using The Human Growth Factors RT2 Profiler PCR Array (Qiagen, USA). The resulting cDNA was mixed with RT2 SYBR Green/ROX PCR Master Mix (Qiagen, USA) and added to 96-well plates. The expression levels of five housekeeping genes (*ACTB*, *B2M*, *GAPDH*, *HPRT*, and *RPLP0*) were used for reference. Expression of the genes *IL6*, *IL8*, *TGFB*, *IGF1*, *VEGFA*, and *CCL2* was analyzed using Qiagen primers (Qiagen, USA). The resulting cDNA was mixed with Reaction mix for RT-PCR with SYBRGreen I (Syntol, Russia) and added to 96-well plates. The expression levels of *GAPDH* and *HPRT* were used for reference. Polymerase chain reaction was performed using the Mx300P system (Stratagene, USA). Normalized gene expression was calculated by the 2^−ΔΔCt^ method^43^.

### Statistical analysis

All values are expressed as the mean ± standard deviation (M ± SD). A minimum of three independent experiments were performed for each assay. Intergroup analysis was performed by nonparametric Mann–Whitney test for independent samples using SPSS 14.0 software. A level of P < 0.05 was accepted as statistically significant.

## Supplementary information

Supplenmentary Figure S1. The effect of TNFα-mediated priming and sµg on ASC secretion.

## Data Availability

The datasets generated during and/or analyzed during the current study are available from the corresponding author on reasonable request.
