# Supplementary material for: Simulated microgravity modulates the mesenchymal stromal cell response to inflammatory stimulation
Source: Sci Rep. 2019 Jun 26;9:9279. doi: 10.1038/s41598-019-45741-8 (PMC6594925; doi:10.1038/s41598-019-45741-8)
Supplement: Supplementary file 1 — Supplenmentary Figure S1. The effect of TNFα-mediated priming and sµg on ASC secretion. [file 41598_2019_45741_MOESM1_ESM.pdf]

Simulated microgravity modulates the mesenchymal stromal cell response to inflammatory stimulation

Andrey Ratushnyy[1], Danila Yakubets[1], Elena Andreeva[1], Ludmila Buravkova[1]*

[1]Lab. of Cell Physiology, Institute of Biomedical Problems of Russia Academy of Sciences, Moscow, 123007, Russia

* buravkova@imbp.ru

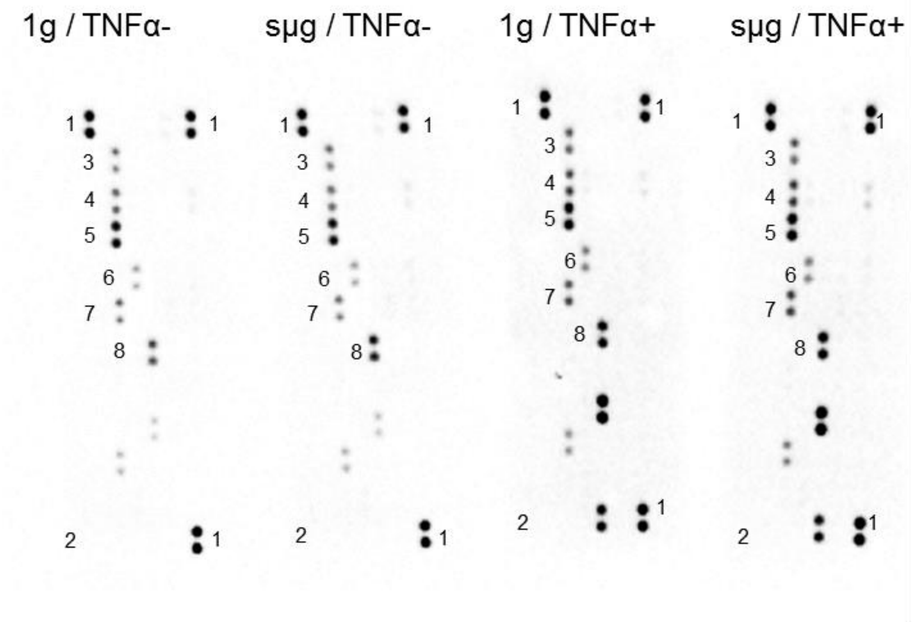

Coordinate	Target/ Control
1	Reference spots
2	Negative control
3	PAI-1
4	PEDF
5	TIMP-1
6	PTX-3
7	TSP-1
8	IGFBP-3

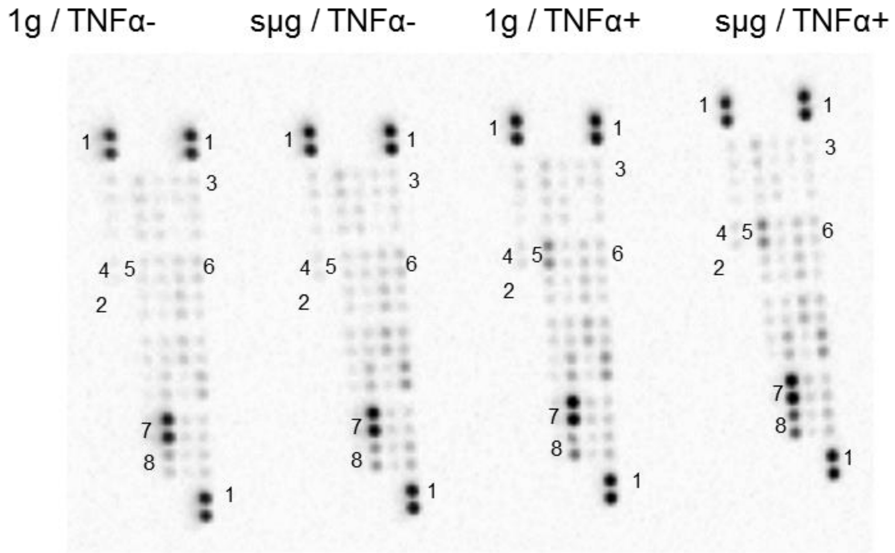

Coordinate	Target/ Control
1	Reference spots
2	Negative control
3	ADAM8
4	uPA
5	MMP-9
6	ADAMATS1
7	MMP-2
8	MMP-3

Supplenmentary Figure S1. The effect of TNFα-mediated priming and sμg on ASC secretion.

To detect secreted proteins, conditioned medium was analyzed using the Proteome Profiler and Proteome Profiler Human Protease Array Kit (R&D, USA) (B) and Human Angiogenesis Array Kit (R&D, USA) (B) according to the manufacturer's instructions. The data were analyzed using Image Lab™ Software Version 5.0 (Bio-Rad, USA).
